# Are people living next to mobile phone base stations more strained? Relationship of health concerns, self-estimated distance to base station, and psychological parameters

**DOI:** 10.4103/0019-5278.58918

**Published:** 2009-12

**Authors:** Christoph Augner, Gerhard W. Hacker

**Affiliations:** IGGMB-Research Institute for Frontier Questions of Medicine and Biotechnology, Salzburg Federal Hospital-University Clinics of the Paracelsus Private Medical University, Muellner Hauptstrasse 48, 5020 Salzburg, Austria

**Keywords:** Base stations, electromagnetic fields, health risks, psychological strain

## Abstract

**Background and Aims:**

Coeval with the expansion of mobile phone technology and the associated obvious presence of mobile phone base stations, some people living close to these masts reported symptoms they attributed to electromagnetic fields (EMF). Public and scientific discussions arose with regard to whether these symptoms were due to EMF or were nocebo effects. The aim of this study was to find out if people who believe that they live close to base stations show psychological or psychobiological differences that would indicate more strain or stress. Furthermore, we wanted to detect the relevant connections linking self-estimated distance between home and the next mobile phone base station (DBS), daily use of mobile phone (MPU), EMF-health concerns, electromagnetic hypersensitivity, and psychological strain parameters.

**Design, Materials and Methods:**

Fifty-seven participants completed standardized and non-standardized questionnaires that focused on the relevant parameters. In addition, saliva samples were used as an indication to determine the psychobiological strain by concentration of alpha-amylase, cortisol, immunoglobulin A (IgA), and substance P.

**Results:**

Self-declared base station neighbors (DBS ≤ 100 meters) had significantly higher concentrations of alpha-amylase in their saliva, higher rates in symptom checklist subscales (SCL) somatization, obsessive-compulsive, anxiety, phobic anxiety, and global strain index PST (Positive Symptom Total). There were no differences in EMF-related health concern scales.

**Conclusions:**

We conclude that self-declared base station neighbors are more strained than others. EMF-related health concerns cannot explain these findings. Further research should identify if actual EMF exposure or other factors are responsible for these results.

## INTRODUCTION

Use of mobile phones has increased dramatically in the last decade. A number of people cannot imagine a world without mobile communication anymore. Coeval with this development and with the increasing presence of mobile phone masts, concerns were raised about possible health risks from electromagnetic fields (EMF) emitted by mobile phones and base stations. The term *Electromagnetic Hypersensitivity (EHS)* was created for symptoms possibly related to EMF. However, the definition and diagnosis remains unclear and controversial, although experts of the WHO (WHO workshop on Electrical Hypersensitivity, Prague, Czech Republic, October 25-27, 2004) defined the new term as Idiopathic Environmental Intolerance (Electromagnetic field attributed symptoms) or IEI-EMF, in order to substitute EHS.[1] According to Röösli and to the results gained in a number of experiments, a linear physiological dose-response relationship between EMF field density and the symptoms seemed to be unlikely for most of the people.[2] On the other hand, Rubin *et al.,* did not see any differences between people with EHS and controls regarding psychopathological diagnoses. Therefore, simple psychopathological explanations are inadequate.[3] Empirical evidence indicates that EHS and EMF-related symptoms, mostly unspecific symptoms, are associated with a variety of psychological and psychobiological mechanisms and parameters. The present study intends to find out how people, who believe that a mobile phone base station is very close to their home, react psychologically and psychobiologically. Furthermore, we wanted to detect how the parameters that are often connected with EHS are related to each other: Self-estimated distance between home and the next mobile phone base station (DBS), daily use of mobile phone (MPU), EMF-health concerns, EHS, and psychological strain and psychobiological stress parameters.

## MATERIALS AND METHODS

A survey was performed with 57 participants, in the run-up of an experiment focusing on EMF emitted by mobile phone base stations and possible health effects, reported elsewhere.[4] The survey consisted of several parts: Asking anamnestic questions on medical history, and the use of four standardized questionnaires, which are, (1) the symptom-checklist SCL-90-R, (2) a standardized questionnaire to assess physical troubles (B-L), (3) a standardized state anxiety questionnaire (STAI), and (4) a well-being questionnaire (MDBF; all German versions) to assess the well-being.[5–8]

Furthermore, we used a self-administered questionnaire designed to assess EMF health concerns, which included two scales (concern of EMF-sources: CSou; concern of EMF-symptoms: CSym), with questions such as *“How strongly are you concerned about your health on grounds of electrosmog from base stations?”*, and so on.

Persons living near base stations (self rated: ≤ 100 meters) were asked to rate their concern about specific symptoms. All others were asked to imagine that they were living next to a base station and to rate their possible concern. As an example, such questions included *“How strongly are you concerned to come down with those symptoms headache?”*, and so on. A response scale from, ‘not at all’ (0) to ‘very strong’ (4), respectively, was used.

Subjects were also asked, if they view themselves as being electromagnetically hypersensitive (0 indicated ‘not at all’ and 4 ‘very strong’).

For our analysis we chose to include data obtained from biochemically assessed parameters, that is, alpha amylase, cortisol, immunoglobulin A, and substance P, quantified in saliva, for the above-mentioned experiments. These variables were measured during an experimental trial after the questionnaire. For our analyses, we used the mean of three test points, 10 minutes, 25 minutes, and 45 minutes, after completing the survey. Saliva was collected using Salivette devices (Sarstedt, Nümbrecht, Germany) that were left in the mouth for five minutes each. Details of preparation and analysis are reported elsewhere [Augner *et al*., manuscript in preparation].

This study was performed in accordance with the guidelines of the expanded Helsinki Declaration[9] and American Psychological Association Ethical Principles of 2002.[10]

For calculation of correlations we used Spearman's rho. For further analysis we dichotomized the sample by DBS in participants by estimating a distance of more than 100 meters (0) and 100 meters or less (1); for independent sample comparisons we calculated by using the Mann-Whitney-U-Test. Data were analyzed using SPSS 16 software (SPSS, Chicago, IL, USA).

## RESULTS

Twenty-two (38.60,%) participants were male and 35 (61.40%) female. The mean age of the sample was 40.72 years (range 18 to 67, SD 12.75). CSou and CSym-scale were highly consistent; the detailed results of consistency and single items have been reported elsewhere.[4]

Regarding DBS, eight (14.04%) people answered that according to their knowledge there was no base station close to their home, 14 (24.56%) rated the distance of a known base station between 100 and 300 meters, 11 (19.30%) between 10 and 100 meters, and three (5.26%) participants rated the distance from their home to the nearest known base station at 10 meters or less. Twenty-one (36.84%) persons chose a ‘do not know’ option. In MPU, two (3.51%) persons answered that they were non-users. Twenty-four (42.11%) participants said they were users, but not on a daily basis, 16 (28.07%) rated themselves as daily users but less than 25 minutes, and 15 (26.32%) answered that they used their mobile phone 25 minutes or more per day.

We correlated the self-estimated distance between home and the next mobile phone base station (DBS) and the psychological and psychobiological parameters, as well as daily mobile phone use (MPU) and those variables. Table 1 shows the results obtained in this correlation analysis. A high DBS score indicated that the participant lived very close to a base station and is probably higher exposed to EMF. MPU was not related to the psychological or psychobiological parameters tested, and there was only one significant correlation with substance P concentration. DBS showed more significant relationships: Although there was no association with health concerns regarding EMFs (sources and symptoms), we detected clear and significant correlations with somatization, anxiety, phobic anxiety (all subscales from the SCL), and with the SCL sum score of PST. The closer the estimated distance to the next mobile phone base station, the higher were the rates of these psychological strain parameters.

**Table 1:** Spearman's correlation between self-estimated distance to base station, daily mobile phone use, and psycho(bio)logical parameters

		CSou	CSym	Amylase	IgA	Cortisol	Substance P
DBS	r	− 0.02	− 0.01	0.26	0.05	− 0.02	− 0.12
	p	0.912	0.961	0.129	0.754	0.905	0.496
MPU	r	0.02	0.06	−0.13	0.21	0.11	0.31*
	p	0.896	0.644	0.325	0.121	0.433	0.022
		Good mood	Alertness	Calmness	EHS	SCL somatization	SCL obsessive compulsive
DBS	r	− 0.30	− 0.12	− 0.30	− 0.08	0.52**	0.29
	p	0.078	0.499	0.073	0.623	0.001	0.082
MPU	r	0.14	− 0.13	− 0.06	− 0.26	0.22	− 0.05
	p	0.306	0.334	0.666	0.055	0.103	0.686
		SCL interpers. sensitivity	SCL depression	SCL anxiety	SCL anger hostility	SCL phobic anxiety	SCL paranoid ideation
DBS	r	0.21	0.20	0.36*	− 0.17	0.50**	−0.05
	p	0.215	0.231	0.031	0.323	0.002	0.775
MPU	r	− 0.04	− 0.02	− 0.02	− 0.12	− 0.03	− 0.20
	p	0.761	0.891	0.880	0.375	0.849	0.135
		SCL psychoticism	SCL PST	State anxiety	Symptoms	DBS	MPU
DBS	r	0.14	0.34*	0.31	0.32	1.00	0.10
	p	0.416	0.044	0.062	0.056	???	0.550
MPU	r	0.01	− 0.13	0.02	0.10	0.10	1.00
	p	0.960	0.348	0.873	0.446	0.550	???

r = Spearman's correlation coefficient; p = level of significance;

** p < 0.01 and

* p <0.05; CSou-health concern to EMF-sources; CSym-health concern to EMF-symptoms; EHS-electromagnetic hypersensitivity; DBS-distance to mobile phone base station; a high score represents close to base station; MPU-daily mobile phone use (minutes); a high score represents frequent use, Good mood – SCL obsessive compulsive, SCL interper. sensitivity – SCL paranoid ideation, SCL psychoticism – MPU.

In our further analysis, we compared the dichotomized sample between base station neighbors (DBS ≤ 100 meters: n = 14) and non-neighbors (DBS > 100 meters: n = 22) [Table 2]. ‘Do not know’ answers were excluded from this calculation.

**Table 2:** Psychological and biological stress parameters and subjective base station distance

	≤ 100 m					> 100 m						
		
	M	SD	P25	Md	P75	M	SD	P25	Md	P75	U	p
CSou	17.46	9.80	9.00	20.00	24.00	19.77	12.21	10.25	20.00	31.50	129.5	0.644
CSym	21.46	18.76	6.50	21.00	29.00	28.29	23.64	7.50	24.00	49.50	116.50	0.478
Amylase*	2.87	1.26	1.64	2.78	4.01	2.04	1.30	1.27	1.53	2.66	87.00	0.043
IgA	247.40	253.41	105.34	141.80	310.50	163.52	86.30	97.83	135.53	232.87	137.00	0.736
Cortisol	3.51	2.66	2.09	2.67	3.78	2.96	1.07	2.06	2.59	3.85	142.50	0.880
Substance P	0.61	0.63	0.25	0.37	0.92	0.66	0.49	0.32	0.54	0.81	100.50	0.340
Good mood	33.93	4.91	30.50	34.50	39.25	37.14	2.87	36.00	38.00	39.00	97.00	0.063
Alertness	33.50	6.58	26.00	35.00	39.25	34.91	5.06	31.75	36.00	40.00	136.50	0.566
Calmness	33.07	5.47	29.50	33.00	39.00	36.27	3.78	35.75	37.00	39.00	95.50	0.056
EHS	0.86	1.10	0.00	0.50	1.25	1.14	1.36	0.00	1.00	3.00	140.00	0.626
SCL somatization*	5.57	4.13	3.00	4.00	9.00	2.91	2.99	0.75	2.00	3.50	82.50	0.019
SCL obsessive compulsive*	5.07	3.36	3.00	4.50	6.25	2.82	1.89	1.00	2.50	4.00	82.50	0.019
SCL interpers. sensitivity	3.79	3.49	1.00	2.50	6.00	2.59	2.22	1.00	2.00	4.00	128.50	0.401
SCL depression	5.29	4.34	2.00	4.50	9.00	2.86	2.46	1.00	2.50	5.25	100.50	0.080
SCL anxiety*	4.21	4.00	1.75	3.00	6.25	1.68	1.25	0.75	2.00	2.25	90.00	0.034
SCL anger hostility	1.71	2.05	0.00	1.00	3.00	1.86	1.78	0.00	2.00	3.00	137.00	0.571
SCL phobic anxiety**	1.64	1.69	0.75	1.00	2.25	0.36	0.66	0.00	0.00	1.00	68.00	0.002
SCL paranoid ideation	2.43	3.01	0.00	1.00	5.25	2.09	1.74	0.75	2.00	3.25	144.50	0.753
SCL psychoticism	2.14	3.06	0.00	1.00	4.00	1.00	1.20	0.00	0.50	2.00	124.00	0.305
SCL PST*	27.21	19.52	11.75	25.00	35.25	16.68	7.64	11.00	18.50	22.00	91.00	0.041
State anxiety*	34.07	6.67	29.25	35.50	38.25	28.27	5.35	24.00	27.50	30.25	80.00	0.016
Symptoms	10.71	12.75	3.00	5.50	13.75	5.68	5.84	1.00	4.00	8.00	114.00	0.192

CSou-health concern to EMF-sources, CSym-health concern to EMF-symptoms, EHS-Electromagnetic Hypersensitivity; Amylase (μU/ml), IgA (μg/ml), cortisol (ng/ml), substance P (ng/ml); M mean, SD standard deviation, P percentile, Md median, U Mann-Whitney-U, *P* means level of significance

** *P* < .01;

* *P* > .05

Self-declared base station neighbors (DBS ≤ 100 meters) had significantly higher concentrations of alpha-amylase in their saliva, higher rates in symptom checklist subscales, SCL somatization, obsessive-compulsive, anxiety, phobic anxiety, and global strain index PST. Furthermore, we found higher values for state anxiety in that group. Therefore, a clear tendency toward higher strain in people subjectively living very close to the base station was found. We did not find any significant differences in EMF health concerns (CSou, CSym) between DBS ≤ 100 meters and DBS > 100 meters.

## DISCUSSION

Obviously there is a need for psychological research in the EMF and health area. Biomedical models alone cannot explain the complex interaction of symptoms, risk perceptions, health concerns, and so on. In our study, we used a number of psychological and psychobiological parameters in order to identify associations with DBS, EMF health concerns, and EHS.

The main finding was that people who rated the distance from their home to the next base station as 100 meters or less had higher scores in psychological strain scales, SCL somatization, obsessive-compulsive, anxiety, phobic anxiety, and global strain index PST. Furthermore, we detected higher concentrations of alpha-amylase in the saliva of those people. There is consistent evidence that the alpha-amylase in saliva is a valuable marker of acute stress reaction.[11] Some studies have also reported elevated amylase in concentrations taken from chronically stressed people.[12] In context with the psychological findings, we hypothesize that higher alpha-amylase concentrations in people rating DBS 100 meters or less, indicates that they are more strained. One could argue that these results support a *Psychosomatic hypothesis*, that is, people fear the base station is what leads to their higher stress level. On the contrary, our results from the concern scales, focusing directly on the health risks from EMF, did not differ between the groups. Therefore, it appears that instead the *Theory of Cognitive Dissonance* of Festinger has a better explanatory value in this context.[13] It seems to be more logical that most people try to play their concerns down when they are aware of living next to a potentially harmful mobile phone base station. Moreover, there is evidence that this effect is mostly observed when individuals have a low or no control of the general situation, for example, neighbors of nuclear power plants.[14]

Recent field studies showed some connections between base station EMF exposure and physical and psychological symptoms.[15–18] In order to test the hypothesis that psychological strain arises from real exposure differences, we invited our participants to a field study carried out subsequently. A technician screened their sleeping rooms to record field densities, primarily for Global System of Mobile Communications (GSM)-EMF. Unfortunately, only 28 (49.12%) persons of the first study participated. In this subgroup, the differences in strain parameters were not significant. For GSM-900 MHz and GSM-1800 MHz in persons rating DBS 100 meters or less, the mean was 856.75 μW/m^2^ (standard error 551.31), and for DBS > 100 meters it was 223.80 μW/m^2^ (standard error 189.40) (p = 0.39, n.s.).[19]

Due to the fact that the subjective estimated distance to the base stations is not a valid indicator for actual exposure and is often criticized, it remains unclear if EMF led to the differences in our group. We conclude that there is more research needed to conclusively study the connection between long-term exposure to mobile phone base stations and psychological strain indicators. Recording of the actual field densities is needed over a longer period of time in rooms where people spend a significant amount of time. Furthermore, self-estimated distances and actual exposure should be correlated.
